# Association between migration status and caesarean section delivery based on a modified Robson classification in China

**DOI:** 10.1186/s12884-021-03708-6

**Published:** 2021-03-17

**Authors:** Ming Liu, Mengqi Xue, Qing Yang, Wenchong Du, Xiaoling Yan, Jing Tan, Tao Duan, Jing Hua

**Affiliations:** 1 The Women and Children’s Health Care Department, Shanghai First Maternity and Infant Hospital, Tongji University School of Medicine, Shanghai, 200000 China; 2 The Obstetrical Department, Shanghai First Maternity and Infant Hospital, Tongji University School of Medicine, P.O. 536 Changle Road, Shanghai, 200042 China; 3 Songjiang Maternity & Child Health Hospital of Shanghai, Shanghai, 200042 China; 4 Department of Psychology, Nottingham Trent University, Nottingham, UK

**Keywords:** Caesarean section rate, Modified Robson classification, Residents and internal migrants, China

## Abstract

**Background:**

China has one of the highest caesarean section (C-Section) rates in the world. In recent years, China has been experiencing a massive flow of migration due to rapid urbanization. In this study, we aimed to differentiate the rates of C-Section between migrants and residents, and explore any possible factors which may moderate the association between migrant status and C-Section rates.

**Methods:**

We conducted a retrospective cohort study in Shanghai, China. All deliveries were classified using the modified Robson Classification. The association between women’s migrant status and C-Section rates was assessed using the Poisson regression of sandwich estimation, after adjusting for possible factors.

**Results:**

Of the 40,621 women included in the study, 66.9% were residents and 33.1% were internal migrants. The rate of C-Section in migrants was lower than that of residents in all subjects (39.9 and 47.7%) and in group 1 subjects (based on the Robson Classification) using a modified Robson Classification. There was an association between migrant status and caesarean delivery on maternal request that was statistically significant (RR = 0.664, *p* < 0.001), but the association was weakened after adjusting for such factors as maternal age at delivery (aRR = 0.774, *p* = 0.02), ethnicity (aRR = 0.753, *p* < 0.001), health insurance (aRR = 0.755, *p* < 0.001), and occupation (aRR = 0.747, *p* = 0.004), but had no significant changes when adjusting for health conditions (aRR = 0.668, *p* = 0.001) and all considering variables (aRR = 0.697, *p* = 0.002). In group 1 subjects, the effect of migrant status on maternal requested intrapartum C-Section was also statistically significant (RR = 0.742, *p* = 0.004).

**Conclusion:**

C-Section rates are lower among migrant women than residents, especially on maternal request. The medical practitioners should further reinforce the management of elective C-Section in resident women.

**Supplementary Information:**

The online version contains supplementary material available at 10.1186/s12884-021-03708-6.

## Background

Caesarean section (C-Section) rates are reported to be associated with higher rates of maternal morbidity and mortality compared to vaginal births [1]. The World Health Organization (WHO) guidelines have deemed C-Section rates of 10–15% as justifiable [2]. C-Section rates of over 15% can lead to increased risks for reproductive health outcomes, which outweigh the benefits of C-Section [3]. However, a large number of countries nowadays have C-Section rates that are much higher than the WHO recommendation [3]. China is one of the countries that have a dramatic increase in caesarean section rates in recent years [4, 5]. A survey from WHO showed that China’s average annual caesarean delivery rate of 46.2% was the highest among nine Asian countries from 2007 to 2008 [6]. Another study involving more than 30 million showed that the C-Section rate in China was 32.7% from 2008 to 2014 [7].

Except for the medical reasons for conducting a C-Section, it has been reported that C-Section rates varied across populations with different socio-demographic status [8–10]. A meta-analysis based on 76 studies reported that there was a difference in caesarean rates between migrants and non-migrants in over 69% of studies [11]. However, this result seemed to conflict with the lower C-Section rates in migrants compared with residents, and there is limited evidence to explain these differences in C-Section rates. Frequently suggested factors that may explain the lower C-Section rates in migrants include younger pregnancy age, healthy immigrant effect, preference for a vaginal birth, and a healthier lifestyle [12, 13]. In contrast, commonly suggested factors for the higher C-Section rates in migrants are poverty, unemployment, low social status, and inadequate prenatal care, among others [14, 15].

China has experienced a massive internal population migration in recent years due to the rapid urbanization process, and the healthcare situation of migrants in China can be more complicated. In China, it can be difficult for migrants to obtain permanent residency in urban areas due to the regulations imposed by the government. In Shanghai, 40.3% of the total population was comprised of internal migrants in 2012 [16]. It has been reported that the internal migrants often face numerous social adversities, [11, 17–19] which may influence their C-Section rates [11, 17–19]. For instance, internal migrants often have a lack of social welfare benefits and/or health insurance, and therefore they are less likely to choose the more expensive C-Section procedure without medical indications. Furthermore, the demography of internal migrants consists of mainly blue-collar workers or housewives, which may be related to the healthy ‘migrant’ effect and the preference for vaginal delivery [20].

To our knowledge, few studies have reported on the C-Section rates between internal migrants and residents in China. Here we conducted a retrospective cohort study in one of the largest maternity hospitals in Shanghai (Eastern Branch of Shanghai First Maternity and Infant Hospital), with approximately 15,000 deliveries per year. We hypothesized that the migrants may experience different C-Section rates compared to residents. Additionally, in order to get a comprehensive picture of the C-Section rates in residents and migrants, we used a modified Robson classification system and divided the subjects into ten groups with distinct obstetric features to assess the respective C-Section rates. The objects of the study were to: (1) assess the C-Section rates in both internal migrants and permanent residents based on the modified Robson ten-group classification system, (2) analyze the effects of migrant status on a caesarean section on maternal request (CSMR), and explore the mediating or moderating factors (especially the socio-demographic factors under Chinese context) which may affect the association between migrant status and C-Section, to provide evidence-based clues in reducing the high rates of C-Section in China.

## Methods

### Participants

We conducted a retrospective cohort study in Eastern Branch of Shanghai First Maternity and Infant Hospital – one of the largest maternity hospitals in China. A total of 41,295 live births (birth weight > 500 g) were recorded between January 1st of 2012 to September 30th of 2014 in the hospital’s electronic database. Most of the variables such as ethnicity, health insurance, occupation, and health condition, were collected retrospectively from the hospital’s electronic medical record registration system. Three variables including previous C-Section, induction of labour, and delivery by C-Section before labour, and the indications of C-Section were manually retrieved from printed medical records at the time of birth. A total of 346 (0.84%) women had to be excluded due to the missing items in electronic medical birth records (such as ethnicity, health insurance, occupation, and health condition). And 328 (0.79%) women were excluded due to missing information about three variables (women with previous C-Section, labour induced, and delivered by C-Section before labour) when we manually collected from printed medical records. Finally, a total of 40,621 live births with complete records were included in the final study.

### Measurement

#### Exposure

We categorized the participants into “residents” and “internal migrants”. Different from the residents, internal migrants refer primarily to migrants within China without local household registration status through the Chinese Hukou system [21, 22]. Internal migrants are most excluded from local educational resources, citywide social welfare programs and many jobs because of their lack of hukou status.

#### Outcomes

The Robson classification system was proposed by examining C-Section within mutually exclusive groups of women with particular obstetric characteristics on the basis of five parameters: obstetric history (parity and previous caesarean section), number of neonates, gestational age (preterm or term), the onset of labour (spontaneous, induced, or caesarean section before the onset of labour), and fetal presentation or lie (cephalic, breech, or transverse). Although the Robson classification scheme has been widely accepted by the international community, it is necessary to revise in several categories for current guidance in obstetric practice. For example, the modified classification [23] separates the intrapartum C-Section after induction of labour and prelabour C-Section because the original Robson classification may miss the important information of the high C-Section rate reduced by the induction of labour which was common in present obstetric population especially in China. Additionally, the modified classification scheme combined the three groups of breech (by parity) and transverse or oblique into one, because the total number of non-cephalic presentation births accounts very small proportion and the vaginal delivery with the non-cephalic presentation is no longer promoted in many countries including China nowadays.

The cesarean section on maternal request (CSMR) is defined as a primary cesarean delivery at maternal request in the absence of any medical or obstetric indication [24–26]. The medical indications for C-Section (MICS) were defined according to the Chinese expert consensus [27], which included the complications of labor and factors increasing the risk associated with vaginal delivery and other complications of pregnancy, pre-existing conditions, and concomitant disease need immediate termination of pregnancy.

#### Other variables

The following socio-demographic factors which may moderate the association between migrant status and C-Section rate were included in our study. We categorized the ethnicity into ‘Han’ and ‘others’ because the majority of the Chinese population belong to Han ethnicity. We divided the maternal age into three age bands of ‘< 20’, ‘20 to 34’, and ‘≥35’. The health insurance was dichotomized into ‘yes’ or ‘no’. We categorized the occupation into ‘Non-managerial position’, ‘Managerial position’, ‘Unemployed’ and ‘Workers (conducting physical labor) or others according to their personal information on the electronic medical record registration system.

There were several adverse health conditions that were not (or not severe enough to be) absolute contraindications to vaginal delivery according to the Chinese expert consensus but considered as moderating factors in our study [27]. Pre-pregnancy BMI is calculated according to the mother’s height and pre-pregnancy weight. Based on the literature, we categorized maternal BMI into ‘< 18.5’(underweight), ‘18.5 to 24.9’(normal weight), and ‘≥25’(overweight) according to the WHO BMI classification, [28] and neonatal birth weight into‘< 2500 g’(low birth weight),‘2500g to 3999g’(normal birth weight) and‘≥4000 g’ (macrosomia). Hypertensive disorder complicating pregnancy was defined as systolic pressure over 140 mmHg or diastolic blood pressure over 90 mmHg. Gestational diabetes mellitus (GDM) was defined as blood glucose levels above 5.1 mmol/L at fasting, 10.0 mmol/L at 1 h or 8.5 mmol/L at 2 h by oral glucose tolerance test. Other maternal complications included maternal renal diseases, HIV infections, prenatal fever. The renal diseases are defined as patients already impaired renal function except for hypertension at the start of gestation. Prenatal fever was diagnosed when the body temperature was higher than 38 °C twice in a row before delivery. Positive HIV (human immunodeficiency virus) antibody throughout pregnancy was diagnosed HIV infections, including AIDS and asymptomatic infections. Other fetal complications included fetal abnormalities and small for gestational age. The fetal malformation is defined as a structural or functional defect caused by abnormal development of an embryo or fetus. A newborn is considered as Small for gestational age (SGA) when birth weight and length below the 10th percentile for gestational age [29].

### Statistical analysis

We calculated the differences of C-Section rate, and the medically indicated and maternal requested C-Section rates between residents and migrants using the Pearson Chi-square test. We also calculated the C-Section rate, and the medically indicated and maternal requested C-Section rates in each group based on the modified Robson classification using Chi-square test. Additionally, differences in demographics, C-Section rates, and indications between migrants and residents were assessed using Pearson Chi-square analysis. Were analyzed using the Pearson Chi-square test and post-hoc comparison.

The associations between women’s migrant status and C-Section rates were estimated when adjusting for possible moderators or mediators (socio-demographics, complications, and other health conditions) or not adjusting for any variables. Relative Risk (RR) was estimated using a modified Poisson regression approach (Poisson regression with a robust error variance). Application of Poisson regression on multi-nominal data has been shown to overestimate for the relative risk [30, 31]. We rectified this using the robust error variance procedure known as sandwich estimation, in our modified Poisson regression [30] using PROC GENMOD in SAS® 9.2 software. A *p*-value of < 0.05 was considered statistically significant.

## Results

Of the 40,621 women included in the final analysis, 66.9% were residents and 33.1% were migrants. There was a difference in C-Section rates between residents (47.7%) and migrants (39.9%) (*p* < 0.001). In addition, there was a difference in CSMR rates between resident and migrant women (20.0% vs. 15.3%, *p* < 0.001), as well as a difference in MICS rates between resident and migrant women (27.6% vs. 24.6%, *p* < 0.001). When we divided the subjects into ten groups according to the modified Robson classification (Table 1), only subjects in group 1 (Nulliparous women with a single cephalic pregnancy, at≥37 weeks of gestation in spontaneous labour) had higher C-Section rates in residents compared to that of migrant women with statistical significance (*p* = 0.005). There were also differences in C-Section rates (with or without medial indications) between residents and migrants in group 1 subjects (*p* = 0.015, Table 1). The CSMR rates in residents were higher than that of migrants in group 7 (Nulliparous women with a single cephalic pregnancy, at ≥37 weeks of gestation, who had caesarean section before labour) and group 8 (Multiparous women, without a uterine scar, with a single cephalic pregnancy at ≥37 weeks of gestation, who had caesarean section before labour) with no statistical significance. We also did not observe the differences in C-Section rates between residents and migrant women in other groups(*p* > 0.05, Table 1).

**Table 1:** The CS rates of Robson Classification by residents and migrants (*n* = 40,621)

	Residents(***n*** = 27,169)			Migrants(***n*** = 13,452)			***p*** ^a^	***p*** ^b, c^
	CS rate %	Rate of CSMR%	Rate of MICS%	CS rate %	Rate of CSMR %	Rate of MICS %		
**Modified Robson Ten-group Classification System**								
1 Nulliparous women with a single cephalic pregnancy, at ≥37 weeks of gestation in spontaneous labour	6.2 (558/8941)	3.1 (279/8941)	3.1 (279/8941)	5.0 (236/4675)	2.4 (112/4675)	2.7 (124/4675)	0.005	0.015
2 Nulliparous women with a single cephalic pregnancy, at ≥37 weeks of gestation, who had labour induced	19.5 (878/4501)	11.2 (504/4501)	8.3 (374/4501)	20.8 (548/2630)	11.1 (291/2630)	9.8 (257/2630)	0.176	0.110
3 Multiparous women, without a uterine scar, with a single cephalic pregnancy at ≥37 weeks of gestation in spontaneous labour	5.1 (61/1199)	1.4 (17/1199)	3.7 (44/1199)	4.6 (43/939)	1.6 (15/939)	3.0 (28/939)	0.588	0.648
4 Multiparous women, without a uterine scar, with a single cephalic pregnancy at ≥37 weeks of gestation, who had labour induced	7.4 (26/350)	2.3 (8/350)	5.1 (18/350)	8.6 (24/278)	1.4 (4/278)	7.2 (20/278)	0.580	0.431
5 Multiparous women, with at least one previous uterine scar with a single cephalic pregnancy at ≥37 weeks of gestation	98.2 (387/394)	0 (0/394)	98.2 (387/394)	96.8 (240/248)	0 (0/248)	96.8 (240/248)	0.237	0.286
6 All women with a single non-cephalic pregnancy, with previous CS	98.7 (1169/1184)	0 (0/1184)	98.7 (1169/1184)	98.6 (562/570)	0 (0/570)	98.6 (562/570)	0.814	0.825
7 Nulliparous women with a single cephalic pregnancy, at ≥37 weeks of gestation, who had caesarean section before labour^d^	100.0 (7419/7419)	51.3 (3803/7419)	48.7 (3616/48.9)	100.0 (2373/2373)	49.6 (1176/2373)	50.4 (1197/2372)	–	0.150
8 Multiparous women, without a uterine scar, with a single cephalic pregnancy at ≥37 weeks of gestation, who had caesarean section before labour ^d^	100.0 (1295/1295)	31.4 (407/1295)	68.6 (888/1295)	100.0 (716/716)	30.2 (216/716)	69.8 (500/716)	–	0.580
9 All women with multiple pregnancies including women with a uterine scar	95.9 (662/690)	31.2 (215/690)	64.8 (447/690)	93.7 (388/414)	33.8 (140/414)	59.9 (248/414)	0.097	0.125
10 All women with a single cephalic pregnancy at < 37 weeks gestation, including women with previous CS	41.6 (498/1196)	17.4 (208/1196)	24.2 (290/1196)	39.4 (240/609)	16.6 (101/609)	22.8 (139/607)	0.384	0.658

^a^ The comparison of CS rate b between resident and migrant women using Chi-square analysis

^b^ The comparison of the ratios of CSMR, MICS and vaginal birth between resident and migrant women using Chi-square analysis in group1 ~ 6 and 9 ~ 10

^c^ The comparison of the ratios of CSMR and MICS between resident and migrant women using Chi-square analysis in group 7 and group 8

^d^ The group is defined as 100% CS rate according to the modified Robson Classification

Table 2 showed the differences in age, ethnicity, occupation, and health conditions between residents and migrant women and among different delivery modes in all subjects and group 1 subjects using the modified Robson Classification. The distributions of detailed health conditions by migrant status and delivery mode were showed in Additional file 1: Table 1. We further analyzed the association between migrant status and C-Section rates (with or without medical indications), and explored the potential factors which may mediate or moderate the above associations (Table 3). In all subjects, migrants were less likely to undergo C-Section when there were no medical indications, compared to residents (RR = 0.664, *p <* 0.001). The protective effects weakened after adjustment for such factors as maternal age at delivery (aRR = 0.774, *p* = 0.02), ethnicity (aRR = 0.753, *p* < 0.001), health insurance (aRR = 0.755, *p* < 0.001), and occupation (aRR = 0.747, *p* = 0.004), but had no significant changes when adjusting for health conditions (aRR = 0.668, *p* = 0.001) and all potential variables which were considered in our study (aRR = 0.697, *p* = 0.002). The migrant status also had protective effects on MICS (RR = 0.776, *p* < 0.001). However, there were no significant changes after adjusting for socio-demographic characteristics (aRR = 0.706 ~ 0.824, each *p* < 0.01) health conditions (aRR = 0.787, *p* < 0.001) and all potential variables (aRR = 0.770, *p <* 0.001).

**Table 2:** Socio-demographics and adverse health conditions by migrant status and delivery mode

	All subjects(***n*** = 40,621)							Subjects of Group 1 based on Robson Classification(***n*** = 13,616)						
	Migrant status			Delivery mode				Migrant status			Delivery mode			
	Residents n(%)	Migrants n(%)	***p***	CSMR n(%)	MICS n(%)	Vaginal Birth n(%)	***p***	Residents n(%)	Migrants n(%)	***p***	CSMR n(%)	MICS n(%)	Vaginal Birth n(%)	***p***
Total numbers	27,169	13,452		7496	10,827	22,298		8491	4675		391	403	12,822	
Maternal age at delivery														
< 20	479 (1.8)	242 (1.8)	< 0.001	180 (2.4)	133 (1.2)	408 (1.8)	< 0.001	185 (2.1)	85 (1.8)	< 0.001	11 (2.8)	7 (1.7)	252 (2)	< 0.001
20–34	23,210 (85.4)	11,772 (87.5)		7316 (97.6)	7467 (69.0)	20,199 (90.6)		8278 (92.6)	4408 (94.3)		380 (97.2)	319 (79.2)	11,987 (93.5)	
≥ 35	3480 (12.8)	1438 (10.7)		0 (0)	3227 (29.8)	1691 (7.6)		478 (5.3)	182 (3.9)		0 (0)	77 (19.1)	583 (4.5)	
Ethnicity														
Han	26,878 (98.9)	12,973 (96.4)	< 0.001	7349 (98)	10,618 (98.1)	21,884 (98.1)	0.809	8849 (99.0)	4527 (96.8)	< 0.001	382 (97.7)	396 (98.3)	12,598 (98.3)	0.713
Others	291 (1.1)	479 (3.6)		147 (2)	209 (1.9)	414 (1.9)		92 (1.0)	148 (3.2)		9 (2.3)	7 (1.7)	224 (1.7)	
Health insurance														
Yes	18,307 (67.4)	6215 (46.2)	< 0.001	5187 (69.2)	5665 (52.3)	13,670 (61.3)	< 0.001	6310 (70.6)	2409 (51.5)	< 0.001	138 (35.3)	236 (58.6)	4523 (35.3)	< 0.001
No	8862 (32.6)	7237 (53.8)		2309 (30.8)	5162 (47.7)	8628 (38.7)		2631 (29.4)	2266 (48.5)		253 (64.7)	167 (41.4)	8299 (64.7)	
Occupation														
Non-managerial position	21,501 (79.1)	9856 (73.3)	< 0.001	5820 (77.6)	8305 (76.7)	17,232 (77.3)	0.312	7034 (78.7)	3473 (74.3)	< 0.001	286 (73.1)	295 (73.2)	9926 (77.4)	0.207
Managerial position	84 (0.3)	138 (1)		43 (0.6)	51 (0.5)	128 (0.6)		34 (0.4)	46 (1.0)		3 (0.8)	2 (0.5)	75 (0.6)	
Unemployed	543 (2)	813 (6)		266 (3.5)	362 (3.3)	728 (3.3)		141 (1.6)	238 (5.1)		11 (2.8)	12 (3)	356 (2.8)	
Workers (conducting physical labor) or others	5041 (18.6)	2645 (19.7)		1367 (18.2)	2109 (19.5)	4210 (18.9)		1732 (19.4)	918 (19.6)		91 (23.3)	94 (23.3)	2465 (19.2)	
Marital status														
Married	27,105 (99.8)	13,410 (99.7)	0.154	7478 (99.8)	10,792 (99.7)	22,245 (99.8)	0.332	8916 (99.7)	4662 (99.7)	0.987	389 (99.5)	401 (99.5)	12,788 (99.7)	0.465
Not married	64 (0.2)	42 (0.3)		18 (0.2)	35 (0.3)	53 (0.2)		25 (0.3)	13 (0.3)		2 (0.5)	2 (0.5)	34 (0.3)	
Adverse health conditions ^a^														
Yes	6102 (22.5)	2674 (19.9)	< 0.001	1695 (22.6)	3188 (29.4)	3893 (17.5)	< 0.001	1493 (16.7)	664 (14.2)	< 0.001	67 (17.1)	111 (27.5)	1979 (15.4)	< 0.001
No	21,067 (77.5)	10,778 (80.1)		5801 (77.4)	7639 (70.6)	18,405 (82.5)		7448 (83.3)	4011 (85.8)		324 (82.9)	292 (72.5)	10,843 (84.6)	

^a^ Having one or more adverse health conditions (these conditions were not (or not severe enough to be) absolute contraindications to vaginal delivery)

**Table 3:** The association between migrant status and CS rates

The adjusted variables	CSMR vs vaginal birth			MICS vs vaginal birth		
	Residents	Migrants RR (95%CI)	***p***	Residents	Migrants RR (95%CI)	***p***
**All subjects (*n*** **= 40,621)**						
Crude ^a^	Ref	0.664 (0.627,0.704)	< 0.001	Ref	0.776 (0.739,0.815)	< 0.001
Adjusted for maternal age at delivery	Ref	0.774 (0.747,0.802)	0.002	Ref	0.824 (0.783,0.867)	< 0.001
Adjusted for ethnicity	Ref	0.753 (0.725,0.779)	< 0.001	Ref	0.774 (0.737,0.813)	< 0.001
Adjusted for health insurance	Ref	0.755 (0.727,0.785)	< 0.001	Ref	0.706 (0.671,0.743)	0.001
Adjusted for occupation	Ref	0.747 (0.726,0.780)	0.004	Ref	0.773 (0.736,0.812)	0.003
Adjusted for adverse health conditions ^**b**^	Ref	0.668 (0.631,0.708)	0.001	Ref	0.787 (0.749,0.827)	< 0.001
Adjusted for all above variables	Ref	0.697 (0.657,0.740)	0.002	Ref	0.770 (0.731,0.812)	< 0.001
**Subjects of Group 1 based on Robson Classification(*n*** **= 13,616)**						
Crude ^a^	Ref	0.742 (0.605,0.910)	0.004	Ref	0.885 (0.699,1.120)	0.308
Adjusted for maternal age at delivery	Ref	0.769 (0.626,0.945)	0.012	Ref	0.873 (0.690,1.105)	0.259
Adjusted for ethics	Ref	0.735 (0.598,0.902)	0.003	Ref	0.886 (0.700,1.123)	0.318
Adjusted for health insurance	Ref	0.730 (0.594,0.896)	0.003	Ref	0.878 (0.693,1.112)	0.281
Adjusted for occupation	Ref	0.737 (0.601,0.905)	0.004	Ref	0.877 (0.693,1.110)	0.275
Adjusted for adverse health conditions ^**b**^	Ref	0.744 (0.607,0.914)	0.024	Ref	0.718 (0.564,0.913)	0.043
Adjusted for all above variables	Ref	0.728 (0.580,0,915)	0.009	Ref	0.767 (0.602,0.978)	0.024

^a^ Not adjusted for any variables

^b^ Having one or more adverse health conditions (these conditions were not (or not severe enough to be) absolute contraindications to vaginal delivery)

In women from group 1 of the modified Robson Classification, the effects of migrant status on CSMR were evident (aRR = 0.742, *p* = 0.004), and changed slightly after adjusting for demographic variables (aRR = 0.730 ~ 769, each *p* < 0.05), health conditions (aRR = 0.744, *p* = 0.024), and all potential variables (aRR = 0.728, *p* = 0.009). However, there were no associations between migrant status and MICS (aRR = 0.885, *p =* 0.308), and was only marginally significant after adjusting for health conditions (aRR = 0.718, *p* = 0.043), and all potential variables (aRR = 0.767, *p* = 0.024). The association between migrant status and C-Section rates after adjusting for adverse health conditions was showed in Additional file 1: Table 2.

## Discussion

The present study sheds light on the effect of migrant status on C-Section rates in urban China based on a modified Robson ten-group Classification [23]. In our study, we observed a lower C-Section rate in migrants than that of permanent residents (39.3% vs. 47.7%) in Shanghai city. The rate of CSMR of migrants was lower than residents in total subjects (15.3% vs 20.0%), and in low-risk women with spontaneous labour (2.4% vs 3.1%). Protective effects of migrant status on C-Section rates has been found on migrant women both with and without medical indications. Interestingly, our results showed that the effect of migrant status on CSMR was moderated by socio-demographic factors (age, ethnicity, health insurance, and occupation). However, in low-risk women with spontaneous labour, the socio-demographics did not mediate or moderate the association between the migrant status and intrapartum C-Section without medical indications.

We observed a lower rate of CSMR in migrants than that of residents. This result was consistent with previous studies, [11, 17–19] which showed that the C-Section rate in migrant women was much lower than that of local residents in several other countries. For example, a prior study involving 9026 women from northern Italy showed that the C-Section rates in Italian immigrants were lower compared to permanent residents (27.4 and 37.1% respectively) [18]. Additionally, when the subjects in our study were divided into ten groups according to the modified Robson Classification, the differences in C-Section rate between migrant and resident women were only seen among nulliparous women with a single cephalic term pregnancy with spontaneous onset of labour (group 1). The present C-Section rates (6.2 and 5.0% in migrants and residents respectively) among nulliparous women with a single cephalic term pregnancy with spontaneous onset of labour (group 1) were slightly higher than the C-Section rates in a neighboring city (3.1–4.3% in Nanjing) [32]. However, further study is needed to explore the related perinatal outcomes of C-Section based Robson Classification.

We further explored the potential moderate factors for the association between migrant status and C-Section rates. Interestingly, we found that the maternal socio-demographics (such as maternal age, ethnicity, health insurance, and occupation) moderated the effects of the migrant status on CSMR after adjusting for these factors. It has been reported that resident women were more likely to be elderly parturient nulliparous compared to their migrant counterparts [33]. Pregnant women over 34 years old were a major risk factor of high C-Section rates according to a previous study [34]. Therefore, the maternity age may moderate the association between migrant status and CSMR. Besides, the moderate effects of ethnicity on the association between migrant status and CSMR were likely caused by the one-child family policy [35]. This policy was only implemented in people of the Han nationality (ethnic majority) by the Chinese government, but not those of ethnic minorities in China. According to a previous study, [36] women under the one-child policy (even those with low-risk pregnancies) were more likely to have selective or elective C-Section in order to prevent their ‘only’ baby from being exposed to the risks that may result from obstetric complications during delivery.

Additionally, it has been reported that health insurance status independently affected elective C-Section [37] . The maternity insurance provided by the government is unfortunately not applicable for migrants in urban China [38]. Thus, these women were more likely to choose a ‘cheaper’ vaginal delivery when there was no medical indication of C-Section present. Moreover, according to our current study and previous studies, [38–40] the percentage of migrant women who did not have a job or only have a low-income job was significantly higher than that of residents. Compared with C-Section delivery, vaginal delivery means a shorter maternal length of stay in hospitals, [41] and paying less for medical care [42]. Therefore, migrants are less likely to select the more expensive operation when there were no medical indications for C-Section. However, it seems reasonable that the above factors (maternal age, ethnicity, health insurance, and occupation) have fewer moderate effects of migrant status on the rate of MICS.

However, in low-risk women with spontaneous delivery (group 1 of Robson Ten-group Classification), the association between migrant status and CSMR was only slightly moderated by maternal demographics and health conditions. The C-Section in women in this group usually occurred during intrapartum period (transfer from spontaneous delivery to C-Section) [43]. A systematic review found that fear of birth during pregnancy may sometimes lead to a request for a maternal requested caesarean birth [44]. Previous studies in China have reported that maternal anxiety, fear of labour pain, or perceived better health for the child and mother, are the main reasons for maternal requested intrapartum C-Section [45–47].

## Conclusions

C-Section rates (especially on maternal request) are lower among migrant women than residents, even in nulliparous women with a single cephalic pregnancy, at≥37 weeks of gestation in spontaneous labour. Our study provided evidence that the resident women should not be neglected for reducing the CSMR, and the medical practitioners should further reinforce the management of elective C-Section. Our study also provided the clues for policymakers to revise some of the strategies (e.g., the type of health insurance) to decrease the maternal requested C-Section. However, the difference is only partly explained by maternal background and health conditions. We did not record all fetal and maternal health conditions, and several socio-demographic factors, such as the migrants’ hometown, a period of residency, and education backgrounds, which may also have an effect on the rates of C-Section [38]. Future studies are needed to investigate these factors in greater detail.

## Supplementary Information

**Additional file 1.**

## Data Availability

Data sets generated and/or analyzed during the current study available from the corresponding author on reasonable request.

## Abbreviations

C-Section: Caesarean sections
MCD: World health Organization (WHO)
RR: Relative risk
CI: Confidence interval
