# Supplementary material for: Association between migration status and caesarean section delivery based on a modified Robson classification in China
Source: BMC Pregnancy Childbirth. 2021 Mar 17;21:215. doi: 10.1186/s12884-021-03708-6 (PMC7971954; doi:10.1186/s12884-021-03708-6)
Supplement: Supplementary file 1 — Additional file 1. [file 12884_2021_3708_MOESM1_ESM.docx]

**Appendix table 1 Detailed adverse health conditions by migrant status and delivery mode^a^**

|  | **All subjects(n=40621)** | | | | | | | |  | **Subjects of Group 1 based on Robson Classification(n=13616)** | | | | | | | |
| --- | --- | --- | --- | --- | --- | --- | --- | --- | --- | --- | --- | --- | --- | --- | --- | --- | --- |
|  | **Migrant status** | | |  | **Delivery mode** | | | |  | **Migrant status** | | |  | **Delivery mode** | | | |
|  | **Residents**  **n(%)** | **Migrants**  **n(%)** | ***p*** |  | **CSMR**  **n(%)** | **MICS**  **n(%)** | **Vaginal Birth**  **n(%)** | ***p*** |  | **Residents**  **n(%)** | **Migrants**  **n(%)** | ***p*** |  | **CSMR**  **n(%)** | **MICS**  **n(%)** | **Vaginal Birth n(%)** | ***p*** |
| Pre-pregnancy BMI^b^ |  |  |  |  |  |  |  |  |  |  |  |  |  |  |  |  |  |
| <18.5 | 3446(12.7) | 877(6.5) | <0.001 |  | 861(11.5) | 1184(10.9) | 2278(10.2) | 0.001 |  | 1149(12.9) | 298(6.4) | <0.001 |  | 47(12.0) | 39(9.7) | 1361(10.6) | 0.028 |
| 18.5~24.9 | 21640(79.6) | 12041(89.5) |  |  | 6067(80.9) | 8940(82.6) | 18674(83.7) |  |  | 7130(79.7) | 4207(90.9) |  |  | 308(78.8) | 332(82.4) | 10697(83.4) |  |
| ≥25 | 2083(7.7) | 534(4) |  |  | 568(7.6) | 703(6.5) | 1346(6) |  |  | 662(7.4) | 170(3.6) |  |  | 36(9.2) | 32(7.9) | 764(6.0) |  |
|  |  |  |  |  |  |  |  |  |  |  |  |  |  |  |  |  |  |
| Hypertensive disorder complicating pregnancy | | |  |  |  |  |  |  |  |  |  |  |  |  |  |  |  |
| No | 26078(96.0) | 13051(97.0) | <0.001 |  | 7006(93.5) | 10226(94.4) | 21897(98.2) | <0.001 |  | 8763(98.0) | 4601(98.4) | 0.094 |  | 371(94.9) | 386(95.8) | 12607(98.3) | <0.001 |
| Yes | 1091(4.0) | 401(3.0) |  |  | 490(6.5) | 601(5.6) | 401(1.8) |  |  | 178(2.0) | 74(1.6) |  |  | 20(5.1) | 17(4.2) | 215(1.7) |  |
|  |  |  |  |  |  |  |  |  |  |  |  |  |  |  |  |  |  |
| Gestational Diabetes Mellitus | | |  |  |  |  |  |  |  |  |  |  |  |  |  |  |  |
| No | 22601(83.2) | 11514(85.6) | <0.001 |  | 6489(86.6) | 8402(77.6) | 19224(86.2) | <0.001 |  | 7770(86.9) | 4162(89.0) | <0.001 |  | 360(92.1) | 318(78.9) | 11254(87.8) | <0.001 |
| Yes | 4568(16.8) | 1938(14.4) |  |  | 1007(13.4) | 2425(22.4) | 3074(13.8) |  |  | 1171(13.1) | 513(11.0) |  |  | 31(7.9) | 85(21.1) | 1568(12.2) |  |
|  |  |  |  |  |  |  |  |  |  |  |  |  |  |  |  |  |  |
| Other maternal complications ^c^ | | |  |  |  |  |  |  |  |  |  |  |  |  |  |  |  |
| No | 26760(98.5) | 13202(98.1) | 0.008 |  | 7301(97.4) | 10656(98.4) | 22005(98.7) | <0.001 |  | 8831(98.8) | 4615(98.7) | 0.791 |  | 374(95.7) | 390(96.8) | 12682(98.9) | <0.001 |
| Yes | 409(1.5) | 250(1.9) |  |  | 195(2.6) | 171(1.6) | 293(1.3) |  |  | 110(1.2) | 60(1.3) |  |  | 17(4.3) | 13(3.2) | 140(1.1) |  |
|  |  |  |  |  |  |  |  |  |  |  |  |  |  |  |  |  |  |
| Newborn’s health conditions | |  |  |  |  |  |  |  |  |  |  |  |  |  |  |  |  |
| Newborn’s birth weight^d^ |  |  |  |  |  |  |  |  |  |  |  |  |  |  |  |  |  |
| <2500g | 1163(4.3) | 705(5.2) | <0.001 |  | 399(5.3) | 766(7.1) | 703(3.2) | <0.001 |  | 76(0.9) | 36(0.8) | 0.017 |  | 3(0.8) | 0(0) | 109(0.9) | <0.001 |
| 2500~3999g | 24478(90.1) | 11870(88.2) |  |  | 7097(94.7) | 8579(79.2) | 20672(92.7) |  |  | 8566(95.8) | 4438(94.9) |  |  | 388(99.2) | 327(81.1) | 12289(95.8) |  |
| ≥4000g | 1528(5.6) | 877(6.5) |  |  | 0(0) | 1482(13.7) | 923(4.1) |  |  | 299(3.3) | 201(4.3) |  |  | 0(0) | 76(18.9) | 424(3.3) |  |
|  |  |  |  |  |  |  |  |  |  |  |  |  |  |  |  |  |  |
| Other fetal complications ^e^ | |  |  |  |  |  |  |  |  |  |  |  |  |  |  |  |  |
| No | 26518(97.6) | 13125(97.6) | 0.830 |  | 7386(98.5) | 10316(95.3) | 21941(98.4) | <0.001 |  | 8792(98.3) | 4607(98.5) | 0.348 |  | 386(98.7) | 366(90.8) | 12647(98.6) | <0.001 |
| Yes | 651(2.4) | 327(2.4) |  |  | 110(1.5) | 511(4.7) | 357(1.6) |  |  | 149(1.7) | 68(1.5) |  |  | 5(1.3) | 37(9.2) | 175(1.4) |  |

^a^ The adverse health conditions were not (or not severe enough to be) absolute contraindications to vaginal delivery

^b^ Maternal BMI were classified into ‘<18.5’(underweight),‘18.5 to 24.9’(normal weight) and ‘≥25’(overweight)

^c^ Other maternal complications included maternal renal diseases, HIV infections, prenatal fever.

^d^ Neonatal birth weight into were classified into‘<2500g’(low birth weight),‘2500g to 3999g’(normal birth weight) and‘≥4000g’(macrosomia).

^e^ Other fetal complications included fetal abnormalities and small for gestational age.

**Appendix table 2 The association between migrant status and CS rates when adjusted for adverse health conditions** ^a^

| **The adjusted variables** | **CSMR vs vaginal birth** | | |  | **MICS vs vaginal birth** | | |
| --- | --- | --- | --- | --- | --- | --- | --- |
|  | **Residents** | **Migrants**  **RR(95%CI)** | ***p*** |  | **Residents** | **Migrants**  **RR(95%CI)** | ***p*** |
| **All subjects (n=40621)** |  |  |  |  |  |  |  |
| Crude ^b^ | Ref | 0.664(0.627,0.704) | <0.001 |  | Ref | 0.776(0.739,0.815) | <0.001 |
| Adjusted for pre-pregnancy BMI^c^ | Ref | 0.673(0.635,0.714) | <0.001 |  | Ref | 0.780(0.742,0.819) | <0.001 |
| Adjusted for hypertensive disorder complicating pregnancy | Ref | 0.671(0.633,0.711) | <0.001 |  | Ref | 0.782(0.745,0.822) | <0.001 |
| Adjusted for gestational diabetes mellitus | Ref | 0.664(0.627,0.703) | <0.001 |  | Ref | 0.787(0.749,0.827) | <0.001 |
| Adjusted for other maternal complications^d^ | Ref | 0.662(0.625,0.701) | <0.001 |  | Ref | 0.817(0.676,0.988) | 0.038 |
| Adjusted for newborn’s birth weight^e^ | Ref | 0.665(0.628,0.704) | <0.001 |  | Ref | 0.775(0.738,0.814) | <0.001 |
| Adjusted for other fetal complications^f^ | Ref | 0.663(0.626,0.703) | <0.001 |  | Ref | 0.775(0.737,0.814) | 0.001 |
|  |  |  |  |  |  |  |  |
| **Subjects of Group 1 based on Robson Classification(n=13616)** |  |  |  |  |  |  |  |
| Crude ^b^ | Ref | 0.742(0.605,0.910) | 0.004 |  | Ref | 0.885(0.699,1.120) | 0.308 |
| Adjusted for pre-pregnancy BMI^c^ | Ref | 0.756(0.617,0.932) | 0.019 |  | Ref | 0.904(0.713,1.147) | 0.407 |
| Adjusted for hypertensive disorder complicating pregnancy | Ref | 0.747(0.609,0.917) | 0.005 |  | Ref | 0.888(0.702,1.125) | 0.325 |
| Adjusted for gestational diabetes mellitus | Ref | 0.740(0.603,0.909) | 0.004 |  | Ref | 0.897(0.708,1.136) | 0.366 |
| Adjusted for other maternal complications^d^ | Ref | 0.756(0.605,0.945) | 0.014 |  | Ref | 0.838(0.676,1.039) | 0.107 |
| Adjusted for newborn’s birth weight^e^ | Ref | 0.747(0.608,0.916) | 0.005 |  | Ref | 0.842(0.663,1.069) | 0.157 |
| Adjusted for other fetal complications^f^ | Ref | 0.758(0.607,0.947) | 0.015 |  | Ref | 0.848(0.684,1.053) | 0.135 |

^a^ The adverse health conditions were not (or not severe enough to be) absolute contraindications to vaginal delivery

^b^ Not adjusted for any variables

^c^Maternal BMI were classified into ‘<18.5’(underweight),‘18.5 to 24.9’(normal weight) and ‘≥25’(overweight)

^d^ These complications included maternal renal diseases, HIV infections, prenatal fever

^e^ Neonatal birth weight into were classified into‘<2500g’(low birth weight),‘2500g to 3999g’(normal birth weight) and‘≥4000g’(macrosomia).

^f^These complications included fetal abnormalities, small for gestational age
